# Estimating natural history of a treatment-eligible type 2 diabetes mellitus population in Scotland using linked primary and secondary care data: Scottish Diabetes Research Network - National Diabetes Dataset

**DOI:** 10.23889/ijpds.v9i5.1673

**Published:** 2024-09-18

**Authors:** Lili Wei, Elaine Butterly, Peter Hanlon, Houra Haghpanahan, Robert Lindsay, Amanda I Adler, Luke A K Blackbourn, Sofia Dias, Katherine Hughes, Stuart McGurnaghan, John Petrie, Naveed Sattar, Laurie A Tomlinson, Nicky Welton, Sarah Wild, Jim Lewsey, David McAllister

**Affiliations:** 1 University of Glasgow; 2 University of Oxford; 3 University of Edinburgh; 4 University of York; 5 Glasgow Royal Infirmary, NHS Greater Glasgow and Clyde; 6 London School of Hygiene and Tropical Medicine; 7 University of Bristol

Healthcare commissioners and those making reimbursement decisions require data on the “natural history” of diseases to estimate quantities such as life expectancy and quality of life. However, such data are commonly acquired from unrepresentative sources like trial placebo arms or case series. We used Scottish Diabetes Research Network-National Diabetes Dataset to characterise life expectancy for people with type 2 diabetes mellitus (T2DM).

Data from >99% of people with diabetes in Scotland are available from primary and secondary care (including from clinical investigations) and can be linked to hospital admission and death records for research purposes. As part of an MRC-funded (MR/T017112/1) systematic review and modelling study (PROSPERO registration CRD42020184174) building on work funded by the Chief Scientific Office for Scotland (CSO HIPS_17_26), we used these linked data to define a treatment-eligible population for three drug classes — SGLT-2 inhibitors, GLP-1 agonists, and DPP-4 inhibitors. Fitting a Gompertz model, we estimated life expectancy for this ‘treatment-eligible’ group.

The treatment-eligible population, compared to the entire Scottish T2DM population, was younger (63.8 (12.1) vs. 66.7 (12.7) year-old), with larger proportions of men (60.6% vs. 56.2%), higher HbA1c levels (67.4 (13.1) vs. 60.2 (15.1) mmol/L), larger proportions prescribed metformin (69.2% vs 55.2%) and higher life expectancy at age 65 (in women 21 vs 19 years and in men 20 vs 18 years).

Using linked data to define and characterise treatment-eligible populations is feasible and may impact decision models. These findings will be useful to those making resourcing decisions and future economic analyses.

